# Heparin: role in protein purification and substitution with animal-component free material

**DOI:** 10.1007/s00253-018-9263-3

**Published:** 2018-08-09

**Authors:** Svenja Nicolin Bolten, Ursula Rinas, Thomas Scheper

**Affiliations:** 1 Institute of Technical Chemistry, Leibniz University of Hannover, Callinstraße 5, 30167 Hannover, Germany; 2 Helmholtz Centre for Infection Research, Inhoffenstraße 7, 38124 Braunschweig, Germany

**Keywords:** Heparin, Heparin-binding proteins, Heparin-protein interactions, Heparin affinity chromatography, Animal-component free

## Abstract

Heparin is a highly sulfated polysaccharide which belongs to the family of glycosaminoglycans. It is involved in various important biological activities. The major biological purpose is the inhibition of the coagulation cascade to maintain the blood flow in the vasculature. These properties are employed in several therapeutic drugs. Heparin’s activities are associated with its interaction to various proteins. To date, the structural heparin-protein interactions are not completely understood. This review gives a general overview of specific patterns and functional groups which are involved in the heparin-protein binding. An understanding of the heparin-protein interactions at the molecular level is not only advantageous in the therapeutic application but also in biotechnological application of heparin for downstreaming. This review focuses on the heparin affinity chromatography. Diverse recombinant proteins can be successfully purified by this method. While effective, it is disadvantageous that heparin is an animal-derived material. Animal-based components carry the risk of contamination. Therefore, they are liable to strict quality controls and the validation of effective good manufacturing practice (GMP) implementation. Hence, adequate alternatives to animal-derived components are needed. This review examines strategies to avoid these disadvantages. Thereby, alternatives for the provision of heparin such as chemical synthesized heparin, chemoenzymatic heparin, and bioengineered heparin are discussed. Moreover, the usage of other chromatographic systems mimetic the heparin effect is reviewed.

**Electronic supplementary material:**

The online version of this article (10.1007/s00253-018-9263-3) contains supplementary material, which is available to authorized users.

## Introduction

The discovery of heparin took place in 1916 when Jay McLean and William Howell isolated a glycosaminoglycan from liver tissues that inhibited blood coagulation. It was called heparin (Greek *hepar*-liver) (McLean 1959; Marcum 1997). In 1928, Howell identified uronic acid as one sugar component in heparin. Seven years later in 1935, Bergstrom and Jorpes discovered glucosamine (GlcN) as the second sugar component in heparin (Jorpes and Bergström 1936). Jorpes determined in 1936 that heparin contains a high amount of sulfo groups which makes heparin one of the strongest known acids. Furthermore, he found out that the sulfo groups are located at the N-residue of GlcN. Several research groups expanded the isolation of heparin first from bovine lung and later from porcine intestine (Liu et al. 2009). At the same time, Jorpes in collaboration with Charles and Scott prepared heparin with acceptable purity for human trials. In 1959, Crafoord and Best showed that heparin treatment prevents postoperative thrombosis. Nevertheless, studies at the Mayo Clinic, Minnesota, revealed that heparin causes side effects such as headaches, fever, nausea, or heparin-induced thrombocytopenia (HIT) (Best 1959). To avoid these side effects, a low-molecular-weight (LMW) heparin fraction was isolated. LMW heparin features defined biological and chemical properties. It shows the desired pharmacological effect, a better bioavailability, a higher therapeutic index, and fewer side effects. Since the 1970s LMW heparin is used for surgery patients to avoid uncontrolled thrombosis (Linhardt and Gunay 1999).

Additionally, since the 1970s heparin is also used in biotechnological processes. It is required as supplement in cell culture media. Furthermore, heparin is used as a specific ligand in affinity chromatography systems to purify protein mixtures due to its interactions with a variety of proteins (Olivecrona et al. 1971; Nordenman and Björk 1977; Shelburne et al. 1977). Nevertheless, heparin is an animal-derived product. Animal products carry the risk of adulterations and contaminations. Based on this risks and the expensive preparation of heparin, a replacement of heparin is of enormous interest in pharmaceutical and biotechnological processes. Hence, this review covers the properties and structure of heparin and the heparin-protein interactions. Strategies for the replacement of heparin in affinity chromatography are also described.

## Structure of heparin

**Fig. 1:**
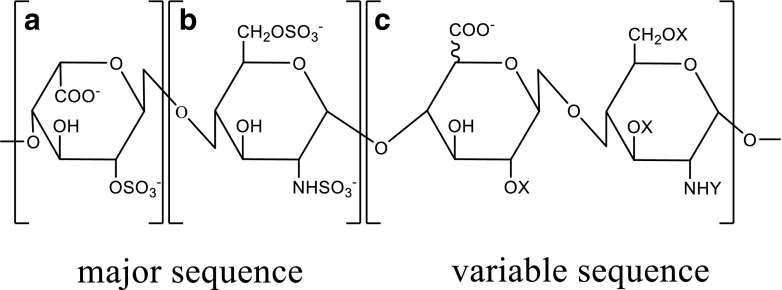
Repeating units of the major and minor disaccharides in heparin. **a** 2-*O*-sulfo-α-l-iduronic acid. **b** 6-*O*-sulfo-*N*-sulfo-α-d-glucosamine. **c** Structural variations of the disaccharide (X = H or SO_3_^−^, Y = Ac, SO_3_^−^, or H). Scheme was adapted from (Capila and Linhardt 2002)

Heparin is part of the family of glycosaminoglycans (GAGs), which are linear polysaccharides characterized by repeating disaccharide units (Perlin and Mazurek 1968; Esko and Linhardt 2009). These repeating units are 1 → 4 glycosidic linked uronic acid and glucosamine residues (Best 1959). Heparin consists of 75–95% of a trisulfated disaccharide repeating unit, 2-*O*-sulfo-α-l-iduronic acid 1 → 4 linked to 6-O-sulfo-*N*-sulfo-α-d-glucosamine (→ 4]IdoA-(2SO_3_)-(1 → 4)-GlcNS-(6SO_3_)-[1 →) (Fig. 1) (Liu et al. 2009).

Nevertheless, there are also structural variations of this disaccharide causing variable heparin sequences. The reason for these variations is that uronic acid consists of l-iduronic acid and d-glucuronic acid, which further can be substituted with a 2-*O*-sulfo group. Furthermore, the glucosamine can also be substituted. Thereby, the amino group can be unsubstituted or substituted with a sulfo or acetyl group. Furthermore, the hydroxyl group on position 3 and 6 can also be substituted with an *O*-sulfo group or still be unsubstituted (Handel et al. 2005). The molecular weight of heparin is in the range of 5 and 40 kDa due to its polydisperse mixture (Handel et al. 2005).

## Biological function of heparin

Heparin is a linear molecule with a helical order which is located intracellularly in mast cells and basophil granules (Mulloy et al. 1993). Its major biological function is the inhibition of the coagulation cascade to maintain the blood flow in the vasculature (Hileman et al. 1998).

It is assumed that l-iduronic acid residues are flexible and responsible for a specific orientation of *O*- and *S*-groups which allows heparin to bind diverse biologically important proteins (Mulloy and Linhardt 2001) such as cytokines, morphogens, growth factors, chemokines, and adhesion molecules (Gandhi and Mancera 2008). Heparin interacts with these heparin-binding proteins via ionic or hydrogen bonds between heparin’s sulfo groups and the amino groups of the protein. Heparin is further involved in different biological processes as cell differentiation, cell growth and migration, inflammation, and pathogen infection (Hileman et al. 1998).

## Provision of heparin

Heparin is a natural product isolated mainly from porcine or bovine tissues. Since the 90s, pharmaceutical heparin is only isolated from porcine intestines because of the bovine spongiform encephalopathy (BSE) crisis. The composition of heparin in the intestines differs. The whole intestine or just the mucosa can be taken for the extraction of heparin. Furthermore, different subspecies of pigs, the habitat, and diet of the pigs have an influence on the composition of heparin (Liu et al. 2009). These factors enhance the already complex composition of heparin. The extraction of pharmaceutical-grade heparin is subject to industrial confidentiality. There are some patents and publications explaining generally used pharmaceutical processes. It starts with the preparation of the tissues and the adjoining extraction of heparin from those. Both steps are at the slaughterhouse and are not subject of current good manufacturing practice (cGMP) conditions. It follows the recovery and purification of raw heparin which are liable under cGMP conditions and can handle with impurities such as other GAGs, heavy metals, bacterial endotoxins, viruses, and bioburden (Liu et al. 2009; Volpi et al. 2012).

## Options of applications of heparin

Heparin is widely used as a therapeutic agent due to its anticoagulation effects to avoid thrombosis. Furthermore, it is injected intravenously to patients during extracorporeal procedures for example during hemodialyses and membrane oxygenation in chirurgic cardiac surgery (Langer et al. 1982).

Besides these extremely important medical applications, heparin is also used in biotechnological research. For example, heparin is used on microarrays for high-throughput analysis of heparin-protein interactions. This process is used for the development of new drugs in antithrombotic use (Park et al. 2008).

Due to Heparin’s specific interactions with various proteins, it is also utilized for protein purification processes (Staby et al. 2005). In this case, heparin is covalently immobilized on a porous bead and acts as a specific affinity ligand. On the basis of its high amount of anionic sulphate groups, it functions also as a cation exchanger (Xiong et al. 2008; Guan and Chen 2014). Protein mixtures can be separated using heparin affinity chromatography columns. Furthermore, the application of heparin affinity chromatography columns often results in concentration of heparin-binding proteins from cell lysates even if they are only present in low concentration (Xiong et al. 2008).

## Advantages and disadvantages of heparin affinity chromatography

The heparin-binding domain of proteins is very important for their biological function. Therefore, the domain is easily accessible for heparin and this takes an advantage in the purification process of recombinant proteins. The heparin affinity chromatography is a very effective and simple method to purify a wide range of proteins. It has a high purification potential and is also easy to handle (Farooqui 1980; Staby et al. 2005; Xiong et al. 2008; Guan and Chen 2014). The heparin affinity chromatography is not dependent on an affinity-tag in contrast to other affinity chromatography systems such as immobilized-metal affinity chromatography (IMAC). Heparin affinity chromatography columns are compatible with oxidizing and reducing agents and chelators (e.g., EDTA) (Farooqui 1980; Xiong et al. 2008).

In the literature, several hundreds of glycosaminoglycan-binding proteins (Table 1) are found (Ori et al. 2011). Different classes of proteins such as the growth factors basic fibroblast growth factor (FGF-2) (Seeger and Rinas 1996), vascular endothelial growth factor (VEGF) (Fiebich et al. 1993), and bone morphogenetic protein-2 (BMP-2) (Vallejo and Rinas 2004), enzymes such as thrombin (Nordenman and Björk 1977), enzyme inhibitors such as antithrombin III (ATIII) (Miller-Andersson et al. 1974), and tyrosine-kinase growth factor receptors such as the FGF receptor (Perderiset et al. 1992) can be purified with a high level of purity.

**Table 1:** Examples of heparin-binding proteins

Nature	Heparin-binding protein	Source
Cytokines/Growth factors		
	BMP-2	Ori et al. (2011)
	FGF-1	Schlessinger et al. (2000)
	FGF-2	Schlessinger et al. (2000)
	Fibroblast growth factor receptor 1 (FGFR1)	Schlessinger et al. (2000)
	FGFR2	Schlessinger et al. (2000)
	Hepatocyte growth factor	Muñoz and Linhardt (2004)
	Heparin binding-epidermal growth factor (HB-EGF)	Aviezer and Yayon (1994)
	Interleukin-1, -2, -3, -4, -5, -7, -8, -10, -12	Koopmann et al. (1999)
	VEGF-A_165_	Robinson et al. (2006)
	Transforming growth factor-β (TGF-β)	Coombe and Kett (2005)
Lipid-binding proteins		
	Annexin V	Capila et al. (2001)
	Apopolipoprotein B (ApoB)	Cardin and Weintraub (1989)
	ApoE	Dong et al. (2001)
Adhesion proteins		
	Fibronectin	Coombe and Kett (2005)
	Vitronectin (Vn)	Cardin and Weintraub (1989)
Chemokines		
	Platelet factor 4 (PF4)	Imberty et al. (2007)
	Regulated on activation normal T cell expressed and secreted (RANTES)	Handel et al. (2005)
Others		
	AT III	Johnson and Huntington (2003)
	Thrombin	Carter et al. (2005)

Heparin affinity chromatography has also some disadvantages. Despite heparin is known for its broad binding specificity to a lot of different proteins, it is not possible to purify a heparin-binding protein from the cell lysate in one single step. It always needs at least one additional step for a complete purification process. Furthermore, heparin is recovered from mucosal tissue of slaughterhouse waste. It is possible that the slaughterhouse waste is infected by animal pathogens such as viruses or prions. Therefore, proteins could be contaminated with these pathogens if they are purified by heparin affinity chromatography (Farooqui 1980).

## Analysis of the heparin-protein interactions

Since the discovery of heparin, several research groups have studied the interactions between heparin and heparin-binding proteins. Cardin and Weintraub started the first experiments to determine the heparin-binding domain (Cardin and Weintraub 1989). They examined four proteins (apolipoprotein B (ApoB), apolipoprotein E (ApoE), vitronectin (Vn), and platelet factor 4 (PF-4)) for their similarities in their heparin-binding domains. Basic clusters with a density of high positive charge were detected. The acidic groups of heparin were electrostatically interacting with these basic clusters. Furthermore, Cardin and Weintraub discovered in these four proteins the two consensus sequences for heparin recognition [-X-B-B-X-B-X] and [-X-B-B-B-X-X-B-X-]. There, X stands for hydropathic residues (Ala, Gly, Ile, Leu or Tyr) and B stands for basic residues (Lys, Arg, and rarely His).

The consensus sequences were used for molecular modeling and were detected in several secondary structural conformations. The modeling experiments indicated that the basic amino acids are outside on one side of the β-strand and the hydropathic residues are inside the protein in the case of the β-strand conformation sequence [-X-B-B-X-B-X]. The sequence [-X-B-B-B-X-X-B-X-] has an α-helix conformation, where the basic amino acids are on one side of the helix and the hydropathic residues face the protein core (Cardin and Weintraub 1989; Torrent et al. 2012). By the help of the molecular modeling studies of Cardin and Weintraub, a third consensus sequence for heparin binding was found in the von Willebrand factor [-X-B-B-B-X-X-B-B-B-X-X-B-B-X-]. This sequence was used to test further proteins for heparin binding. Unfortunately, the consensus sequences could not be identified in any other heparin-binding protein (Sobel et al. 1992).

By further studies, it was detected that the conformation of the heparin-binding site is not important for the interactions but rather the distance of ~ 20 $\dot{A}$ between the basic amino acids (mainly arginines) (Margalit et al. 1993). In this gap, a pentasaccharide would fit. Furthermore, it was suggested that heparin is wrapping around the heparin-binding sides of the protein. This coiled-coil-like structure can cause changes in the protein conformation.

A few years later, Hileman et al. proposed another consensus sequence for heparin binding while using X-ray and NMR for the screening of acidic and basic fibroblast growth factor (FGF-1 and FGF-2) and transforming growth factor β-1 (TGFβ-1). The pattern is [-T-X-X-B-X-X-T-B-X-X-X-T-B-B-] where B and X have the same meaning as already mentioned above, and T stands for a turn (Hileman et al. 1998).

Torrent et al. determined a structural rather than a sequence motif comprising one polar (Asn, Gln, Thr, Tyr or Ser and fewer Arg or Lys) and two cationic residues (Arg or Lys) which was called “CPC Clip Motif” (Torrent et al. 2012). There, C stands for cationic residues and P stands for polar residues. The cationic and polar residues identified a clip-like structure in which heparin can be placed (Mosier et al. 2012; Dempewolf et al. 2013; Green et al. 2013). Within this motif, the cationic amino acids were responsible for the major interactions with heparin and the polar amino acid for the fine tuning. For this study, they screened 20 heparin-binding proteins for structural motifs that interact with heparin via electrostatic interactions, hydrogen bonding, and van der Waals bonds. It was found that the basic amino acids Arg and Lys were necessary for the hydrogen-bonding and electrostatic interactions with heparin. They confirmed the research of Cardin-Weintraub that Lys and Arg as well as Ala, Gly, Ile, Leu, or Tyr were essential in heparin binding. Otherwise, they could not detect a Cardin Weintraub motif in every heparin-binding protein they analyzed. Further, the CPC clip motif was also found in proteins which are indicated not to bind heparin (Torrent et al. 2012; Pulido et al. 2017). In Table 2, all motives are summarized.

**Table 2:** Comparison of the patterns of heparin-binding proteins

Pattern	Bound proteins	Source
[-X-B-B-X-B-X] and [-X-B-B-B-X-X-B-X-]	ApoB, ApoE, PF-4, Vn	Cardin and Weintraub (1989)
[-X-B-B-B-X-X-B-B-B-X-X-B-B-X-]	von Willebrand factor	Sobel et al. (1992)
[-T-X-X-B-X-X-T-B-X-X-X-T-B-B-]	FGF-1, FGF-2, TGFβ-3	Hileman et al. (1998)
Cation-Polar-Cation (CPC-Clip motif)	20 heparin binding proteins (e.g., FGF-1, FGF-2)	Torrent et al. (2012)

*X* hydropathic residues like Ala, Gly, Ile, Leu or Tyr, *B* basic residues like Lys, Arg or fewer His, *T* turn, *C* cationic residue like Lys or Arg, *P* polar residue like Asn, Gln, Thr, Tyr or Ser and fewer Arg or Lys

Up to now, it is not completely explored which parts of heparin bind proteins and the other way round. The best way to characterize the interactions between proteins and heparin is with the help of crystal structures.

## Crystal structures of heparin-protein complexes

A couple of research groups are dealing with the protein-heparin interactions, whether it is for medical or biotechnological applications. Four proteins with a strong affinity to heparin are in the main focus. These include different types of biologically active proteins such as the cytokines FGF-1 (Zhu et al. 1993; DiGabriele et al. 1998) and FGF-2 (Faham et al. 1996; Pellegrini et al. 2000; Schlessinger et al. 2000), the serin protease thrombin (Li et al. 2004; Carter et al. 2005), and the glycoprotein ATIII (Mulloy and Linhardt 2001; Li et al. 2004; Coombe and Kett 2005; Xu and Esko 2014). These proteins differ not only in their protein class but also in their pI and molecular weight. The different research groups could not use heparin itself for their studies since it is a very heterogeneous molecule. Therefore, heparin-simulating molecules are used. The interactions between these heparin mimicry and the listed biologically active proteins are shown in Table 3.

**Table 3:**
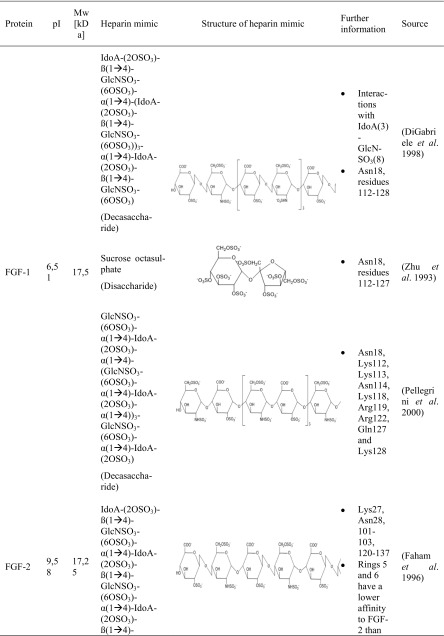

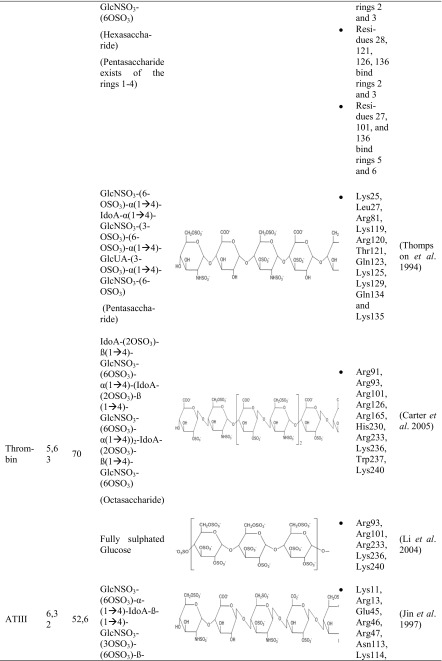

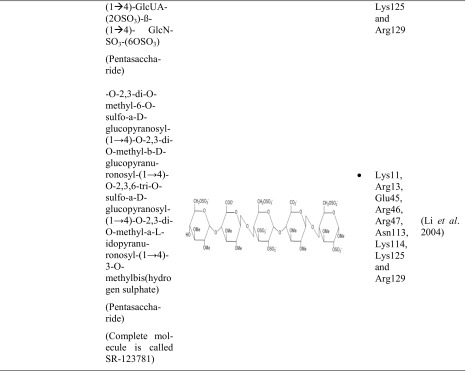
Characteristics of protein-heparin interactions

*GlcNSO*_*3*_ = *N*-sulfo-α-d-glucosamine, *GlcNSO*_*3*_*-(6OSO*_*3*_*)* = 6-*O*-sulfo-*N*-sulfo-α-d-glucosamine, *GlcNSO*_*3*_*-(3OSO*_*3*_*)-(6OSO*_*3*_*)* = 3, 6-*O*-sulfo-*N*-sulfo-α-d-glucosamine, *IdoA* = α-l-iduronic acid, *IdoA-(2-OSO3)* = 2-*O*-sulfo-α-l-iduronic acid, *GlcUA-(3-OSO3)* = 3-*O*-sulfo-α-d-glucuronic acid

### Crystal structure of the fibroblast growth factors-heparin complex

Fibroblast growth factors (23 members) regulate cellular processes such as cell growth and cell differentiation. Their signal transduction system is omnipresent and important in the developmental process in embryos and the homeostasis in adults (Ye et al. 2001). The interaction with heparin enhances their bioactivity (DiGabriele et al. 1998).

FGF-1 was crystallized with a fully sulfated heparin decasaccharide to identify the interactions between FGF-1 and heparin (Table 3). Most of the interactions between FGF-1 and heparin were ionic caused by the basic residues of FGF-1 and the acidic sulfate and carboxylate groups of heparin (Table S1). Furthermore, FGF-1 interacts with five to six monosaccharides of opposite sites of the heparin fragment. Hence, no interactions are exactly alike and variations are in the interactions of FGF-1 and heparin (DiGabriele et al. 1998).

The results complied with other crystal structures in which sucrose octasulfate (SOS) (Zhu et al. 1993) or un/sulfated oligosaccharides were used to identify the sugar-binding side of FGF-1 (Ornitz et al. 1995; Faham et al. 1996).

It was further detected that Lys112 and Lys118 interacted twice with each sugar ring of sucrose octasulfate (Table 3). The furanose was bound two to three times by Arg116 and Arg122 (Zhu et al. 1993).

A study with a decasaccharide consisting of five glucosamine and iduronic acid disaccharides endorsed these results (Table 3). It was detected that one decasaccharide bound two FGF-1 proteins. The binding site of the first FGF-1 consisted of polar and basic residues. FGF-1 interacted with six monosaccharides (IdoA-1 to GlcN-4). The second FGF-1 turned 120° compared to the first FGF-1 and interacted with five monosaccharides (GlcN-1 to GlcN-3). As a result, the second FGF-1 interacted with heparin by different residues. Lys105, Trp107, Lys112, Arg119, Pro121, and Arg122 presented the heparin-binding domain. The heparin-binding sites differed in the residues Lys105, Trp107, and Pro121 (Pellegrini et al. 2000).

Different research groups studied the interactions between heparin and FGF-2. FGF-2 was crystallized with two homogenous fragments of heparin-a tetra- and a hexasaccharide (Table 3). The heparin fragments consisted of a concatenation of the same disaccharide, 2-*O*-sulfo-α-l-iduronic acid 1 → 4 linked to 6-*O*-sulfo-*N*-sulfo-α-d-glucosamine (→ 4]IdoA-(2OSO_3_)-β(1 → 4)-GlcNSO_3_-(6OSO_3_)[1 →) (Fig. 1). The hexasaccharide had three disaccharides and the tetrasaccharide two disaccharides. Both heparin fragments bound similar residues. Most of the specific interactions between heparin and FGF-2 were mediated by negatively charged groups (Table S2). FGF-2 had a higher binding affinity to the hexasaccharide which is probably caused by the larger contact region. It can be concluded that the residues which bound the second and third ring of both saccharides belong to the main heparin-binding site whereas the residues which bound the fifth and sixth ring belong to the minor heparin-binding site (Faham et al. 1996).

The pentasaccharide GlcNSO_3_-(6-OSO_3_)-α(1 → 4)-IdoA-α(1 → 4)-GlcNSO_3_-(2-OSO_3_)-(6-OSO_3_)-α(1 → 4)-IdoA-(3-OSO_3_)-α(1 → 4)-GlcNSO_3_-(6-OSO_3_) (Table 3) was also used to identify the heparin-binding domain of FGF-2 and determined similar. Arg81 bound indirectly heparin through a water molecule. Further experiments showed that Lys125 is the most important residue for heparin-FGF-2 interactions. It was detected that the heparin-protein interactions are also mediated by hydrophobic interactions, hydrogen bonding, and van der Waals forces (Thompson et al. 1994; Torrent et al. 2012). Accordingly, not sulfated heparin fragments like carboxyl and hydroxyl groups can also interact with protein residues (Ornitz et al. 1995).

Recapitulating, the heparin-binding domain of FGF-1 and FGF-2 featured similarities which could be attributed to the fact that their structure identity is higher than 50% (Galzie et al. 1997). Furthermore, Zhu detected that the residues Lys112 and Lys118 from FGF-1 are the analogous residues of Lys119 and Lys125 from FGF-2. These residues are important in the protein-SOS-interactions (Zhu et al. 1993).

### Crystal structure of the thrombin-heparin complex

The heterodimer thrombin is a serine protease which cleaves soluble fibrinogen. This leads to the development of polymerogenic fibrin and causes the fibrin clot and therefore blood coagulation. Thrombin is important in the process of wound healing and inflammations. The normal blood flow is maintained when thrombin is inhibited by antithrombin (AT) or heparin through an irreversible method (Carter et al. 2005).

The crystal structure of thrombin discloses two cationic patches on the surface. These are identified as two anion-binding exosites. Exosite I is the fibrinogen recognition, exosite and exosite II the commonly believed heparin-binding exosite.

To identify the heparin-thrombin interactions, an octasaccharide heparin fragment was used. The saccharide consisted of a linkage of four disaccharides (IdoA-(2OSO_3_)-GlcNS-(6SOSO_3_))_4_ (Table 3). Each of the two heparin molecules was crystallized with two thrombin monomers. All four thrombin monomers had a similar conformation. Two monomers interacted the most with six monosaccharides of heparin. The other two interacted with five monosaccharides. The experiments showed that the sulfate groups of heparin were mostly interacting with thrombin (Table S3). These residues are negatively charged and interacting with basic amino acids of thrombin (Arg, Lys, His). Recapitulating, these kinds of interaction are of ionic nature (Carter et al. 2005).

Further studies identified also Lys235 as a binding residue (Xu and Esko 2014). By mutagenesis, different research groups detected the importance of this single basic residue. Following ranking shows the importance of the different residues of thrombin in heparin-binding: Arg93 > Lys236 > Lys240 > Arg101 > Arg233 (Sheehan and Sadler 1994; Gan et al. 1994; Tsiang et al. 1997; Carter et al. 2005).

Three glucose units covered with ten sulfate groups were also used to study the protein residues interacting with heparin (Table 3). The results endorsed the research of Carter. Arg93, Arg101, Arg233, Lys236, and Lys240 were involved in the interactions with the heparin mimic. Arg93 interacted three times with the heparin mimic, Lys236 twice, Arg101 and Arg233 once, and Lys240 could form three interactions (was not fully modeled) (Table S4) (Li et al. 2004).

### Crystal structure of the ATIII-heparin complex

ATIII is a serpin and inhibits serine proteases such as thrombin. It is one of the most important inhibitors of the blood coagulation and circulates with a low reactivity but in a high concentration in the bloodstream. Heparin supports and catalyzes the activity of ATIII by interaction (Mulloy and Linhardt 2001).

It was shown that the unique pentasaccharide sequence GlcNSO_3_-(6-OSO_3_)-α(1 → 4)-IdoA-α(1 → 4)-GlcNSO_3_-(3-OSO_3_)-(6-OSO_3_)-α(1 → 4)-GlcUA-(2-OSO_3_)-α(1 → 4)-GlcNSO_3_-(6-OSO_3_)-OH bound ATIII with a high affinity (Table 3). The third monosaccharide (glucosamine) was modified with a 3-*O*-sulfate. This modification is very unusual but probably important for the high affinity to ATIII (Lindahl et al. 1984). Studies revealed that ATIII bound heparin with a 1000-fold higher affinity than heparin without this modification (Rosenberg 1978). This pentasaccharide is very rare and is present in just one third of heparin chains (Casu et al. 1981; Riesenfeld et al. 1981).

In further studies, this pentasaccharide was also used to determine the interactions between heparin and ATIII (Table 3). It was shown that the residues Lys11, Arg13, Asn45, Arg46, Arg47, Glu113, Lys114, Lys125, and Arg129 were involved in the interactions with the heparin fragment (Jin et al. 1997).

Another working group explored also the interactions between heparin and ATIII with a similar pentasaccharide (Table 3). The results reinforced the previous studies and are summarized in Table S5. The interactions between heparin and ATIII were based on electrostatic interactions and hydrogen bonds (Li et al. 2004).

### Conclusion of the crystal structure studies

The four examples represent three different protein classes additionally with a different molecular weight and an individual pI. In each case, the heparin-binding domain consisted of a cluster of basic amino acids (Arg, Lys). These clusters are not based on the amino acid sequence but probably on their secondary structure. Mostly, some surrounded amino acids form one part of the heparin-binding domain. The entire domain exists of at least two parts across the whole protein.

The analysis of the heparin fragments reveal that the sulfate residues are mostly involved in the interactions with the proteins. It is secondary whether it is an *O*-sulfated or *N*-sulfated residue. The carboxyl and hydroxyl groups are less involved in the interactions.

Recapitulating, the analysis of the heparin-binding domain emerges that the heparin-binding domain is not made of a definite pattern. The heparin-protein interactions are not only electrostatic. It is a combination of different interactions which leads to the specificity of heparin to the heparin-binding proteins.

## Strategies to replace animal-derived heparin

Over $3 billion of pharmaceutical heparin is sold every year. The demand is about 100 metric tons per year and is expected to further increase. However, the provision of the animal tissue is limited and hence also for heparin. Furthermore, heparin as an animal-derived material involves the risk of contaminations such as viruses and prions. Additionally, in 2008, heparin was contaminated with oversulfated GAGs from pigs in China. This heparin contamination crisis caused the death of 100 people in the USA alone (Guerrini et al. 2008). In addition, the heparin production is subject to strict quality control and the validation of effective GMP implementations. This results in a huge interest on the development of an alternative production of heparin from non-animal origin. These include the chemical and chemoenzymatical synthesis or the bioengineering of heparin. Furthermore, the heparin affinity chromatography could be replaced by already-established chromatography methods.

### Chemical synthesized heparin

**Fig. 2:**
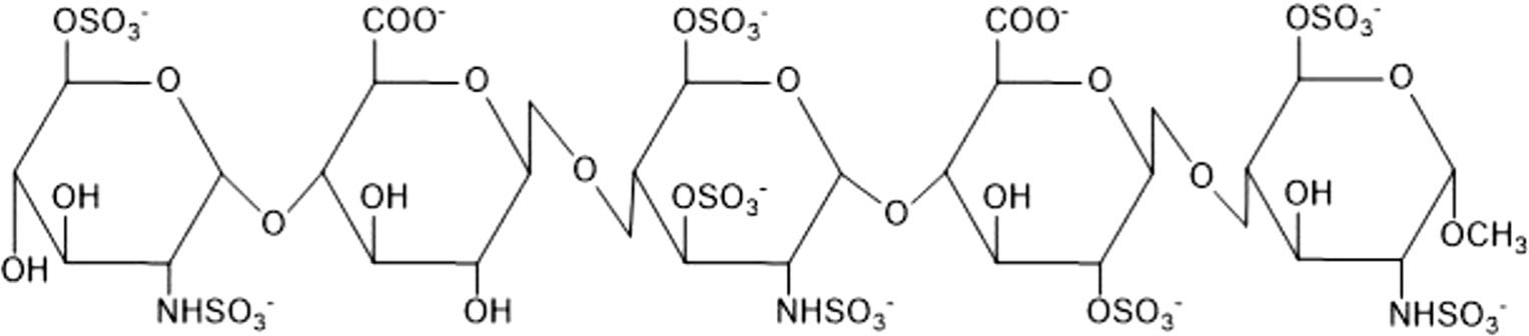
Structure of the pentasaccharide fondaparinux

In 2002, a synthetic pentasaccharide of heparin went on sale. The substance is called Fondaparinux (Fig. 2) and binds the coagulation factor AT-III which inhibits the formation of thrombin as well as the thrombus growth and disturbs the coagulation cascade. The name of the drug is Arixtra (marketed within the US by Dr. Reddy’s Laboratories). However, the production of this drug is very expensive regarding the multistep chemical synthesis, the expensive materials, and the low yield. This led to a restricted clinical application (Petitou and Van Boeckel 2004; Liu and Linhardt 2014). Furthermore, this drug is specific just for the binding of AT-III and does not possess other significant pharmacological characteristics of heparin. Moreover, Arixtra has a longer half-life than heparin and eliminates the effect by the antagonist protamine which could lead to uncontrolled bleeding (Buller et al. 2007; Zulueta et al. 2013).

### Chemoenzymatic heparin

**Fig. 3:**
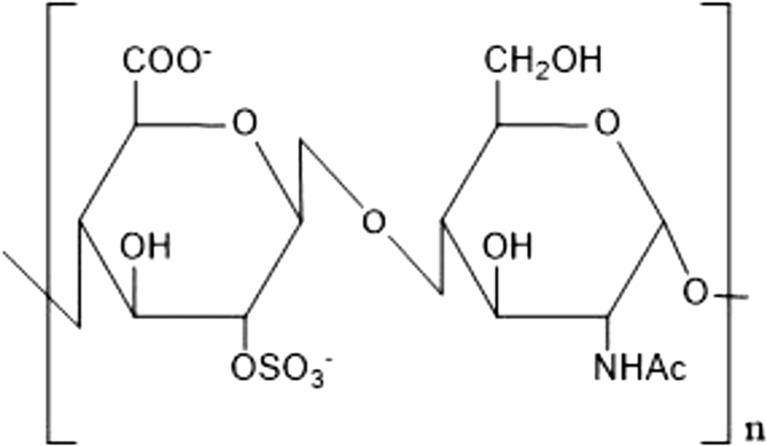
Structure of the heparin precursor heparosan

The chemoenzymatic synthesis of heparin is based on its biosynthetic pathway. It is a heparin analogous pentasaccharide which is not extracted from intestines. That pentasaccharide binds ATIII and leads to anticoagulation. The “neoheparin” is produced by a combination of biosynthetic and chemoenzymatic modifications of a polysaccharide produced from the strain *Escherichia coli* K5 (*E. coli* K5). This strain produces naturally the unsulfated precursor of heparin ([GlcAβ-(1 → 4)GlcNAcα(1 → 4)]_n_, the so-called Heparosan (Fig. 3)) (Lindahl et al. 2005; Zulueta et al. 2013).

Heparosan is *N*-deacetylated and *N*-sulfated in some steps and further modificated by recombinant C5 epimerases and O-sulfotransferases. It is a six-step process with a 60% recovery rate of the polysaccharide, and the yield is at gram scale (Lindahl et al. 2005). Disadvantages of this process are the limitation of the start material for an up-scaling. Furthermore, the specificity of the enzymes could vary the composition of “neoheparin” (Masuko and Linhardt 2012; Fu et al. 2016).

### Bioengineered heparin

A promising idea is the production of pharmaceutical heparin in mammalian cell systems such as the Chinese hamster ovary (CHO) cell system (Baik et al. 2012). The CHO cell system has been intensively studied for the production of pharmaceutical products such as recombinant enzymes, hormones, and monoclonal antibodies. CHO cell systems have also the ability to produce non-protein pharmaceuticals such as heparin. These systems are characterized by their natural glycosylation of CHO cell proteins and are safe regarding potential biological contaminations and easy to scale up (Baik et al. 2012).

CHO cells produce heparan sulfate (HS) which consists of the same disaccharides as heparin but less sulfated. Nevertheless, HS and heparin share the same biosynthetic pathway and HS is such a heparin, an anticoagulant but with a lower activity (Robinson et al. 1978). For the heparin synthesis, HS can be used as precursor. CHO cells provide most of the enzymes which are needed for the production of bioengineered heparin. They do not express mouse heparin sulfate 3-*O*-sulfotransferase 1 (Hs3st1) und *N*-deacetylase/*N*-sulfotransferase (NDST2) (Xu et al. 2012; Fu et al. 2016). Both enzymes are involved in the synthesis of the AT-binding pentasaccharide and *N*-sulfation of GlcNAc. Therefore, they are relevant for the anticoagulant properties of heparin (Sugahara and Kitagawa 2002). For the production of heparin in CHO cells, the genes of Hs3st1 and NDST2 have to be transfected into CHO host.

Disadvantageous is that the engineered heparin does not have the same trisulfated structure which is common in animal-derived heparin. The expressed heparin showed a higher level of anticoagulant activity than in not transfected cell lines. Nevertheless, the activity of bioengineered heparin compared to animal-derived heparin was inadequate (Baik et al. 2012).

### Replacement of heparin in affinity chromatography systems

Several working groups established already other methods to purify heparin-binding proteins with animal-component free processes. The recombinant human BMP-2 was often used as model protein.

Sharapova et al. published one method to purify BMP-2 without heparin affinity chromatography. A strong cation exchange chromatography resin was used.

The protein was produced in inclusion bodies of *E. coli*. BMP-2 in inclusion bodies was first denatured and solubilized. The purification of the protein was performed with the refolding solution in one single step by the cation exchange column S-sepharose FastFlow. BMP-2 was biological active and had a purity of 95% (Sharapova et al. 2010).

A further method to purify BMP-2 without heparin showed Guo et al. In this study, BMP-2 was purified by a two-step hydrophobic interaction chromatography (HIC). Both steps were performed by a Phenyl Sepharose Fast Flow High Sub column.

BMP-2 was produced in inclusion bodies of *E. coli*. The protein was solubilized and refolded prior to the two purification steps. There was no need of a buffer exchange since HIC starts at a high salt concentration. The first chromatography step is used as concentration step of BMP-2 from the refolding solution. The second step functions as purification step. The pooled elution fractions of the first chromatography step were applied to the column. This time, a higher NaCl concentration and 5% *N*,*N*-dimethylformamide (DMF) were added. DMF disrupts the interactions between protein and ligand and leads to an elution of BMP-2 in the flow-through during rebalancing step. The protein was purified with more than 95% and biological active. The purification yield was higher than 20% (Guo et al. 2012).

Rane et al. (2013) purified BMP-2 by a weak cation exchange resin and polished by a size exclusion column. The protein was also produced in inclusion bodies of *E. coli*. After solubilization, BMP-2 was on-column refolded on a weak cation exchange resin with carboxylic acid as functional group. After a buffer exchange, BMP-2 was polished by a size exclusion column. The purity of the biological active protein was higher than 90%. Disadvantageous is the low yield of 14% (Rane et al. 2013).

In addition, Gieseler et al. published a method to purify BMP-2 by mixed-mode membrane chromatography (MMC). This membrane adsorber consists of reinforced cellulose membrane packed with cation-exchange groups and hydrophobic ligands. The forming interactions can be hydrophobic and ionic. Furthermore, this MMC is salt tolerant which is advantageous for purifying salt containing BMP-2 refolding mixture.

BMP-2 was produced in inclusion bodies of *E. coli*. After denaturation and solubilization of the inclusion bodies, the refolding solution was directly applied to the MMC. The purity of the biological active protein was higher than 90%. A disadvantage of this method is the low recovery rate of rhBMP-2 caused by buffer exchange or dialysis (Gieseler et al. 2017).

These publications demonstrated that animal-component-free methods exist at least in the case of BMP-2. All methods resulted in a biological active protein and a purity of more than 90%. Best results were performed by the strong cation exchange chromatography (Sharapova et al. 2010) and by HIC (Guo et al. 2012) with a purity of at least 95%. For comparison, BMP-2 has a purity of almost 100% after heparin-affinity chromatography (Ruppert et al. 1996; Quaas et al. 2018).

Further model proteins were FGF-1 and FGFR from the cell lysate where Batra et al. showed a way to purify these proteins by a weak cation exchanger. They used an Amberlite cation exchange resin (IRC) 50. The compounds of this resin are copolymerized methacrylic acid and divinylbenzene. Therefore, the main functional groups are carboxyl groups and the minor groups are methyl and phenyl groups. The forming interactions can be hydrophilic and hydrophobic and thus provide affinity and hydrophobic binding sites for the analyte. This method is timesaving and inexpensive compared to heparin affinity chromatography.

FGF-1 and FGFR were both recombinant expressed by *E. coli*. While FGF-1 remains soluble after cell growth, FGFR is present in inclusion bodies. After the chromatography step, the purity of both proteins was about 98%. The total yield of FGF-1 was 30 mg/L which is similar to the yield after using heparin-affinity chromatography (32 mg/L). Additionally, FGF-1 showed biological activity after purification. FGFR had a yield higher (24 mg/L) than after conventional purification methods (20 mg/L) (Batra et al. 2011).

Additionally, Pizarro et al. used a further method to purify a heparin-binding protein without heparin. The recombinant human vascular endothelial growth factor A-165 (VEGF) was produced in inclusion bodies of *E. coli*. After solubilization and refolding, VEGF was purified by three chromatography steps.

The first chromatography step functions as a capture step of VEGF. The protein was applied to a multimodal cation exchanger (Capto™ MMC) because of the high pH of the refolded VEGF. This column has the characteristics of a weak cation exchanger but is featured with further ligand structures. Additionally to the electrostatic interactions, hydrogen bonding and hydrophobic and thiophilic interactions can also be formed.

For the next step, a strong cation exchange column (SP Sepaharose High Performance) was used to remove the host cell impurities and product variants. In the last step, the sample was applied to a hydrophobic interaction chromatography column (Phenyl Sepharose™ 6 Fast Flo-low substitution) to polish the protein. Host cell impurities and aggregates below target levels were removed in this step. The purity was higher than 99% and the protein was also bioactive (Pizarro et al. 2010).

## Concluding remarks and future prospects

Heparin binds specifically to a variety of biotechnological important proteins. This makes heparin affinity chromatography an important method for purifying several proteins. It is comfortable to use and does not require any additional protein tags like other affinity chromatography methods. However, heparin chromatography is limited by the unregulated production of heparin out of slaughterhouse waste. As a result, it is inapplicable for GMP-compliant purifications. As heparin is very heterogeneous and no definite pattern of the protein-binding region is known, heparin is still not replaced in affinity chromatography systems. Nevertheless, in this review, two possibilities for the heparin affinity chromatography with non-animal-derived heparin are discussed.

The first opportunity is the production of animal-component free heparin by chemical or chemoenzymatic synthesis or by metabolic engineered CHO cells. However, these bioengineered heparins have been employed only for pharmaceutical reasons and structure analysis. Although promising to our knowledge, there is no study about using bioengineered heparin for purification methods up to now.

The second opportunity is the utilization of already known chromatography techniques to replace heparin chromatography for purifying proteins. At least six research groups examined already established chromatography methods to purify heparin-binding proteins. Single-step purification (IRC or MMC) and multi-step purification (HIC or a combination of Capto™ MMC, CEX, and HIC) showed a similar purification grade compared to the purification by heparin affinity chromatography. The special feature of heparin is its specificity to various proteins. The interactions are not only electrostatic but also a combination of different interactions (van der Waals forces, hydrogen bonds, hydrophobic, and thiophilic interactions). These characteristics were utilized for the development of new purification strategies. It must be noted that most of the protein samples were purified from refolded inclusion bodies. These proteins are of a higher purity than soluble expressed proteins. Therefore, these methods should be first tested for their applicability for other heparin-binding proteins especially for soluble proteins.

## Electronic supplementary material

ESM 1 (PDF 11 kb)
