# Supplementary material for: Heparin: role in protein purification and substitution with animal-component free material
Source: Appl Microbiol Biotechnol. 2018 Aug 9;102(20):8647–60. doi: 10.1007/s00253-018-9263-3 (PMC6153649; doi:10.1007/s00253-018-9263-3)
Supplement: Supplementary file 1 — (PDF 11 kb) [file 253_2018_9263_MOESM1_ESM.pdf]

Supplementary material

Intended Journal

Applied Microbiology and Biotechnology

Mini-Review

Heparin: Role in Protein Purification and Substitution with Animal-Component Free Material

Authors

Svenja Nicolin Bolten[1], Ursula Rinas[1,2], Thomas Scheper[1]

[1] Institute of Technical Chemistry, Leibniz University of Hannover, Callinstraße 5, 30167 Hannover, Germany

[2] Helmholtz Centre for Infection Research, Inhoffenstraße 7, 38124 Braunschweig, Germany

Correspondence

Prof. Dr. Thomas Scheper (scheper@iftc.uni-hannover.de; +49 511762 2509)

Tables

Table S1 Analysis of the heparin mimic (hexa-saccaride) involved in the interactions with FGF-1 (DiGabriele *et al*. 1998)

Monosaccharide	Monosaccharide residue	Protein residue
GlcNSO$_3$-(6OSO$_3$)	2-NHSO$_3^-$	Asn18, Lys118, Gln127, Lys128
	6-OSO$_3^-$	Asn18, Asn114, Arg122,
IdoA-(2OSO$_3$)	2-OSO$_3^-$	Asn18, Lys118, Gln127,
	3-OH	Asn18
	6-COO$^-$	Lys112, Lys113, Lys128

Table S2 Analysis of the heparin mimic (hexa-saccaride) involved in the interactions with FGF-2 (Faham *et al*. 1996)

Monosaccharide	Monosaccharide residue	Protein residue
GlcNSO$_3$-(6OSO$_3$)	2-NHSO$_3^-$	Lys27, Asn28, Asn102, Arg121, Lys126
	3-OH	Lys27
IdoA-(2OSO$_3$)	2-OSO$_3^-$	Gln135, Lys136, Ala137
	6-COO$^-$	Arg121, Lys136

Table S3 Analysis of the heparin mimic (hexa-saccaride) involved in the interactions with thrombin (Carter *et al*. 2005)

Monosaccharide	Monosaccharide residue	Protein residue
GlcNSO$_3$-(6OSO$_3$)	2-NHSO$_3^-$	Arg91, Arg93, Arg101, Lys236, Trp237, Lys240
	3-OH	Arg233
	6-OSO$_3^-$	His91, Arg93, Arg101, Arg165, His230, Lys240
IdoA-(2OSO$_3$)	2-OSO$_3^-$	His91, His93, Arg101, Arg126, Lys236, Lys240
	6-COO$^-$	Lys236

Table S4 Analysis of the interaction of glucose covered with sulphates with thrombin (Li *et al*. 2004)

Monosaccharide	Monosaccharide residue	Protein residue
Fully sulphated glucose	2-OSO_3	Lys236, Lys240
	3-OSO_3	Arg93
	6-OSO_3	Arg93, Arg101, Arg233

Table S5 Analysis of the interaction of glucose covered with sulphates with antithrombin (Li *et al*. 2004)

Monosaccharid residue	Protein residue
2-OSO_3^-	Arg13, Arg47, Glu113, Lys114
3-OSO_3^-	Asn45, Lys114, Lys125
OMe	Arg47
6-OSO_3^-	Asn45, Lys125, Arg129
6-COO^-	Lys11, Asn45, Arg46, Arg47
